# Bio-Active Peptides from Marine Sources: Mechanistic Insights into Immune Regulation, Microbiota Modulation, and Intestinal Barrier Protection

**DOI:** 10.3390/ijms262110508

**Published:** 2025-10-29

**Authors:** Farman Ali, Dailin Li, Yunpeng Su, Lixue Chen, Xiaoxin Cheng, Xu Zheng, Jun Mao

**Affiliations:** 1 College of Basic Medical Sciences, Dalian Medical University, Dalian 116044, China; falm@dmu.edu.cn (F.A.); dailinli1104@163.com (D.L.); suyp@dmu.edu.cn (Y.S.); chengxiaoxin@dmu.edu.cn (X.C.); 2 College of Pharmacy, Dalian Medical University, Dalian 116044, China; chenlx01@dmu.edu.cn; 3 Liaoning Key Laboratory Cancer Stem Cells, College of Basic Medical Sciences, Dalian Medical University, Dalian 116044, China

**Keywords:** marine peptides, gut microbiota, immune modulation, intestinal barrier, IBD, natural products

## Abstract

Natural bioactive chemicals sourced from marine species have attracted growing interest due to their immunomodulatory, antioxidant, and gut microbiota-regulating characteristics. These chemicals, especially peptides, offer therapeutic approaches for addressing inflammation, immunological dysfunction, and intestinal barrier disturbance, which are frequently observed in conditions such as inflammatory bowel disease (IBD). This review centers on current discoveries about marine-derived peptides from octopus, sea conch, and scallop. These substances have demonstrated a considerable ability to restore intestinal integrity, regulate immune cell function, reduce pro-inflammatory cytokines, and rebalance dysbiotic gut microbiota. We consider several in vivo scenarios, encompassing dextran sulphate sodium (DDS)-induced colitis and cyclophosphamide-induced immunosuppression. These compounds raise the expression of tight junction proteins (including ZO-1 and occludin), boost the production of mucin, and encourage the growth of good bacteria such as *Lactobacillus* and *Lachnospiraceae*. Their effects are mechanistically associated with the inhibition of critical inflammatory pathways (e.g., Nuclear factor-κB (NF-κB), Toll-like receptor 4 (TLR-4)) and the modulation of both innate and adaptive immune responses. These versatile bioactives can serve as dietary supplements or complementary therapies for gastrointestinal and cancer-related issues. This review emphasizes the therapeutic potential of marine peptides, concentrating on gut–immune–microbiota interactions, as well as exploring future avenues for clinical translation and drug development

## 1. Introduction

Marine ecosystems, which encompass more than 70% of the Earth’s surface, are the epicenter of biodiversity on our planet. These habitats are home to a vast range of organisms, from minute phytoplankton to colossal marine mammals. All of these organisms have adapted to thrive in the ocean’s unique and often harsh environment. This evolution has led to the development of bioactive molecules, such as peptides and proteins, that have different structural, functional, and variety characteristics than those found in plants on land [1,2]. Protein-derived bioactive peptides are defining as short polymers of amino acids purported to possess multifaceted health benefits, exerting a considerable influence on various metabolic and physiological functions [1]. Marine-derived bioactive peptides, typically consisting of 3 to 40 amino acid residues, are not just fascinating in terms of their potential, but also in terms of their versatility. Peptides that are originally inactive in their parent protein sequences gain biological activity when they are released through digestion in the stomach, enzymatic processing, or fermentation. Their participation in many biological functions depends on their amino acid sequence, content, and molecular weight. Peptides, due to their capacity to engage with cellular receptors enzymes and ion channels, are crucial for the regulation of several physiological processes. The adaptability of peptides derived from marine sources renders them a viable field for research and prospective use [3,4]. Marine organisms including fish, seaweed, shellfish, microalgae, crustaceans, cephalopods, and mollusks, as well as their by-products, constitute vital reservoirs of proteins and bioactive peptides. These marine-sourced proteins and peptides have garnered considerable scientific attention owing to their diverse range of biological activities, such as antioxidant, antihypertensive, antidiabetic, anticoagulant, antibacterial, anti-inflammatory, anticancer, and immunomodulatory effects. Their multifunctional nature renders them particularly advantageous for applications within the food, pharmaceutical, and cosmetic sectors [1,5,6]. In preclinical studies, peptides obtained from marine organisms, including octopus, sea conch, and scallop, have exhibited the ability to enhance the immune system and influence gut microbiota [7,8,9,10].

Marine-derived peptides and proteins, with their distinctive structures shaped by the ocean’s environmental conditions, hold great promise. These compounds often show higher stability, bioavailability, and sustainability, and lower toxicity than their counterparts on land. The ability of marine species to adapt to very salty water, high pressure, varying oxygen levels, and different temperatures, underlines the potential of their biochemical adaptations. This potential inspires optimism and hope for the creation of molecules that work better [1,11]. Recent advancements in extraction and analytical techniques have improved our ability to identify and describe bioactive peptides derived from marine sources. Proteins can be broken down into these bioactive peptides through a process called hydrolysis. This can be achieved using traditional methods as well as more modern approaches, including alkaline or hydrolysis, solvent extraction, enzymatic hydrolysis, microbial fermentation, and emerging methods such as ultrasound-assisted extraction, pulsed electric field application, pressurized liquids extraction, and sub-critical water extraction [12]. Enzymatic hydrolysis is employed in the food and pharmaceutical sectors because of the potential residue of toxic organic solvents or compounds from alternative processes in the final goods [13]. Marine-derived peptides, derived from protein breakdown, often contain impurities that can reduce their effectiveness. To maximize the potential of these molecules, it is essential to employ advanced purification methods. This purification phase is a critical step, especially for the commercial development of peptides, as it ensures their standardized quality and biological activity. To achieve these goals, several sophisticated methods are routinely utilized, including ultrafiltration, gel filtration chromatography, ion-exchange chromatography, and reverse-phase high-performance liquid chromatography (RP-HPLC). For instance, immunomodulatory peptides derived from *Stolephorus chinensis* have been successfully isolated through an optimized protocol involving g ultrafiltration, ion-exchange chromatography, and RP-HPLC. Such advanced purification strategies significantly enhance peptides’ purity and potentiate their functional properties, thereby making them suitable for incorporation into pharmaceuticals and functional food products [14].

Chronic intestinal disorders, including IBD, which encompasses ulcerative colitis (UC) and Crohn’s disease (CD), as well as gastrointestinal damage caused by chemotherapy, pose significant global health challenges. These conditions are characterized by chronic inflammation, immune system dysfunction, compromised integrity of the intestinal barrier, and an imbalance in the gut microbiota [15]. Current medications, such as corticosteroids, immunosuppressants, and biologics, can manage symptoms well, but they often have problems such as side effects, a higher risk of infection, and lower effectiveness over time [16]. Natural chemicals, especially bioactive peptides obtained from marine species, have surfaced as potential replacements or adjuncts to traditional medicines [17]. Preclinical investigations utilizing models such as dextran sulphate sodium (DSS)-induced colitis and cyclophosphamide (CTX)-induced immunosuppression have demonstrated the capacity of these peptides to restore gut barrier integrity by reinstating the expression of tight junction proteins (ZO-1, occludin) and enhancing mucin production. Furthermore, their administration is associated with a marked reduction in pro-inflammatory cytokine levels (TNF-α, IL-1β) and a favorable modulation of the gut microbiota, notably promoting the proliferation of beneficial genera such as *Lactobacillus* and *Lachnospiraceae* [7,8,9,10,18]. Mechanistically, their actions entail the manipulation of critical signaling pathways, including NF-κB and TLR-4, as well as the regulation of innate and adaptive immune responses. This diverse impact on immunology, intestinal barrier integrity, and microbial equilibrium indicates therapeutic promise for IBD and chemotherapy-induced mucosal damage [7,8,9,10].

This review summarizes the contemporary research on peptides originating from marine sources, emphasizing their functions in controlling gut inflammation, augmenting intestinal barrier integrity, regulating immunological responses, and reinstating microbial equilibrium. We consolidated information from experimental models, encompassing DSS-induced colitis and cyclophosphamide (CTX)-induced immunosuppression. We emphasized the medicinal potential of these substances. We further examine their significance to IBD and gastrointestinal health, providing insights on clinical use and therapies based on natural products.

## 2. Sources and Extraction of Bioactive Compounds

Even though there are a lot of different types of marine settings, research on bioactive proteins and peptides, defined as macromolecules with specific physiological functions such as antimicrobial, antioxidants, or antihypertensive activities, has predominantly focused on a limited subset of organisms. These primarily include fish, various algal species (e.g., red, green, and brown algae, alongside spirulina), crustaceans (e.g., crabs, crayfish, lobster, prawns and shrimp), marine bacteria, and specific invertebrate groups such as mollusks, sponges, echinoderms, and cnidarians [19]. Collagen has been obtained from several marine organisms, such as fish, sea cucumbers, mollusks, sponges, crabs, seaweed, and jellyfish [20]. Shellfish, including prawns, crabs, crayfish, and lobster, represent a valuable reservoir of protein and bioactive peptides. The opioid peptides derived from shellfish are considered a particularly compelling subject of research [21]. Shellfish-derived peptides have been characterized as potent angiotensin-converting enzyme (ACE) inhibitors, facilitating blood pressure reduction through the obstruction of ACE activity. Notable examples encompass valyl-tyrosie (VY) isolated from shrimp (*Litopenaeus vannamei*) protein hydrolysates, isoleucyl-prolyl-proline from oysters (*Crassostrea gigas*), and isoleucyl-leucine-proline from crab (*Portanus trituberculatus*) protein hydrolysates. Furthermore, shellfish serve as a source of various immunomodulatory proteins and peptides, including hemocyanins present in mollusks and crustins in crustaceans, which are known to augment phagocytic activity and induce cytokine production [22]. Investigations utilizing spontaneously hypertensive rat models have revealed the considerable antihypertensive potential of gelatin hydrolysates obtained from marine sources such as sea cucumbers, skate skin, jellyfish, and squid skin. This is particularly significant given that hypertension is a modifiable risk factor for cardiovascular disease. Fish species, such as Atlantic salmon, cod, herring, hoki, pacific whiting, Pollack, snappers, and sole, suggesting their promising potential for conferring health benefits [23].

Bioactive peptides derived from marine sources have attracted considerable attention for their versatile roles in modulating immune responses, repairing intestinal barriers, and reshaping the gut microbiota. Their effectiveness depends on both their biological origin and the extraction or hydrolysis methods used, which influence their molecular composition, structure, and bioactivity [24]. Marine organisms including the octopus (*Octopus vulgaris),* sea conch *(Rapana venosa*), and scallop (*Patinopectin yessoensis*) are abundant sources of low-molecular-weight peptides. These peptides are generally extracted through enzymatic hydrolysis using proteases such as pepsin, trypsin, or alcalase. The process involves washing, homogenizing, and hydrolyzing the raw material under controlled temperature and pH conditions, followed by centrifugation, filtration, and lyophilization to produce peptide-rich hydrolysates. Peptides from octopus, obtained via enzymatic hydrolysis, have been shown to modulate immune function and gut microbiota in immunosuppressed mice. Scallop peptides, derived through enzymolysis, exhibited immunomodulatory activity, increased tight junction protein expression, and improved gut microbial profiles in CTX-treated mice. After hydrolysis, peptide identification and activity are often characterized using SDS-PAGE, liquid chromatography-mass spectrometry (LC-MS), and Fourier-transform infrared spectrometry (FTIR) to determine size, composition, and functional groups. Functional characterization of peptides is crucial for understanding their biological activities. Key techniques include FTIR spectroscopy for identifying functional groups (O-H, C-H, C-O-159 C bonds), LC-MS or HPLC for determining molecular weight and peptide sequences or monosaccharide composition, SEM for visualizing surface morphology, and 16S rRNA 161 sequencing for assessing changes in gut microbial communities’ post-treatment [7,8,9,10,18]. In addition to these analytic approaches, the physicochemical and biological properties of marine-derived peptides determine their overall activity and functionality. These peptides generally exhibit low molecular weights (below 3 kDa) and contain hydrophobic amino acids such as leucine, valine, and phenylalanine, which enhance membrane permeability and radical-scavenging ability. The presence of aromatic and sulfur-containing residues (tryptophan, tyrosine, cysteine, and methionine) contribute to their strong antioxidant and metal-chelating capacities. Many marine peptides also possess ACE-inhibitory and immunomodulatory properties, enabling them to regulate blood pressure and modulate immune responses. Moreover, their amphipathical and cationic nature facilitates interaction with cell membrane and microbial surface, providing additional antimicrobial and cytoprotective effects. Together, these structural and compositional characteristics define the diverse bioactivities and stability profile of marine bioactive peptides, supporting their potential as nutraceutical and therapeutic agents.

## 3. Bioactivities of Marine-Derived Peptides

**Figure 1:**
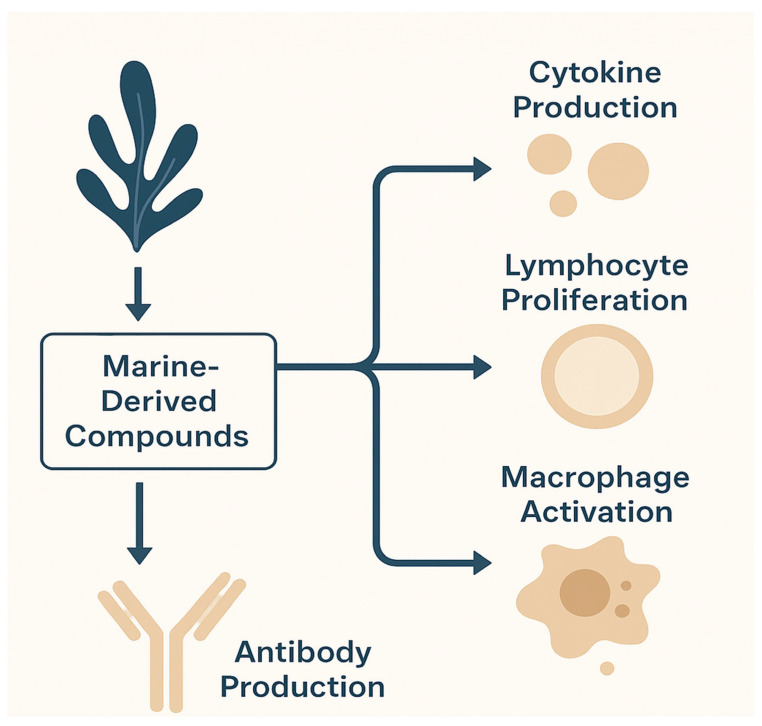
Bioactivities of marine-derived peptides. Marine-derived peptides and proteins exert diverse biological functions, including antioxidant, immunomodulatory, intestinal-barrier-protective, gut-microbiota-regulating, and anticancer activities. These compounds act through multiple mechanisms such as free radical scavenging, modulation of immune cell signaling, restoration of epithelial integrity, and maintenance of microbial homeostasis. Collectively, these bioactivities provide a therapeutic basis for the prevention and management of chronic diseases, including inflammatory bowel disease (IBD).

Marine organisms, including fish, algae, crustaceans, bacteria, and invertebrates, serve as principal reservoirs for bioactive proteins and peptides. These macromolecules offer several distinct advantages, such as high metabolic compatibility, an absence of religious dietary prohibitions, and a reduced risk of transmitting terrestrial animal-borne pathogens. They exhibit a broad spectrum of biological activities, encompassing antioxidant, antihypertensive, antidiabetics, anticoagulant, antibacterial, anti-inflammatory, antithrombotic, anticancer, and immunomodulatory effects. In addition to religious and ethical considerations, the use of marine derived peptides is governed by international food and health safety standards. According to FAO/WHO and EFSA guidelines, all bioactive peptides intended for human consumption must comply with purity, non-toxicity, and allergenicity assessments to ensure consumer safety. Potential risks associated with marine allergens, heavy-metal residues, and microbial contamination are evaluated during production and purification stages. Moreover, the Good Manufacturing Practice (GMP) and Hazard Analysis and Critical Control Point (HACP) frameworks are recommended to measure collectively ensure that marine peptides meet global nutraceutical and pharmaceutical safety standard before commercialization. A summary of the biological activities associated with marine-derived peptides is presented in Figure 1.

### 3.1. Antioxidant Activity

Oxidation constitution is a fundamental component of metabolic pathways in animals and humans. However, this process generates ROS and free radicals, which can disrupt cellular homeostasis and precipitate oxidation stress. Such stress possesses the capacity to inflict damage upon cellular components and is implicated in the pathogenesis of numerous chronic conditions, including cardiovascular disease, stroke, arteriosclerosis, diabetes, and cancer [25]. Additionally, the oxidation of lipids by ROS is a significant problem for both the food business and consumers. It causes food to go bad and makes compounds that are dangerous and taste bad. Lipid peroxidation is bad for both the food business and people’s health [26]. Marine-derived proteins and peptides exhibit considerable antioxidant potential, which is attributed to their ability to neutralized free radicles, chelate pro-oxidant metal ions, and augment endogenous antioxidant defense systems. The primary mechanism underlying their antioxidant activity involves the donation of a hydrogen atom or an electron from the antioxidant molecules, thereby neutralizing free radicals and mitigating their associated adverse effects [27]. Peptides with antioxidant properties obtained from sea cucumbers (*Holothuria leucospilota*) are proposed as a natural source of antioxidant compounds for applications in the pharmaceutical and food sectors. Additionally, protein hydrolysates obtained from *Stichopus japonicus* exhibit protective properties against oxidative stress. In H_2_O_2_-exposed Vero cells, administration of the hydrolysate led to a reduction in intracellular ROS, increased cell viability, and attenuated apoptotic damage. Correspondingly, in a zebrafish embryo model subjected to H_2_O_2_-induced oxidative stress, the hydrolysate treatment significantly lowered cell mortality, which was concomitant with a marked decreased in ROS and lipid peroxidation levels [28]. These preparations also demonstrated considerable ferrous ion chelating capacity and notable reducing power. The antioxidant efficacy of these hydrolysates was further corroborated in biological systems, evidenced by the inhibition of low-density lipoprotein (LDL) oxidation and the protection Of DNA from oxidation damage [29]. Antarctic krill (*Euphausia superba*) antioxidant peptides exhibit significant reducing capacity, provide effective protection against H_2_O_2_-induced plasmid DNA damage, and they possess the ability to reduce lipid peroxidation [30]. Guanghua et al. [31] show that peptides made from the mantle type V of the pearl oyster *(Pinctada martensii*) work better as antioxidants than type I collagen from tilapia (*Oreochromis niloticus*) scales. This finding shows that *P. martensii* is a better natural antioxidant for the food processing industry. Zhang and colleagues [32] showed that antioxidant peptides isolated from Mytilus coruscus enhanced cell viability and mitigated morphological damage in human umbilical vein endothelial cells (HUVECs). Substantial research efforts have been directed towards the investigation of bioactive peptides derived from fish hydrolysates, with a particular emphasis on those originating from fish processing by-products [33]. Bashir et al. identified novel antioxidant peptides within the muscle protein hydrolysates of mackerel (*Scomber japonicas*). The peptide sequence ALSTWTLQLGSTSFSASPM demonstrated the most potent radical scavenging activity, whereas the peptides LGTLLFIAIPI exhibited the highest superoxide dismutase (SOD)-like activity [34]. Researchers have investigated novel antioxidant peptides derived from tilapia (*Oreochromis niloticus*) skin gelatin hydrolysates. Zhang et al. established that the peptides Glu-Gly-Leu and Tyr-Asp-Glu-Tyr possess significant hydroxyl radical scavenging capacity [35]. The study examined the valorization of biomass waste from tuna processing, using fermentation to discover four novel antioxidant peptides: YENGG, EGYPWN, YIVYPG, and WGDAGGYY. These peptides demonstrated significant efficacy in scavenging hydroxyl radicals. This effect was further potentiated through Neutrase-associated microwave hydrolysis, a phenomenon likely attributable to the resultant reduction in peptide size and the increased presence of hydrophobic amino acid residues [36]. Freitas et al. [37] extracted a novel heptapeptide, TCGGQGR, from hydrolysates of mackerel by-products. It possesses significant antioxidant properties, rendering it beneficial for various applications in the food industry [38]. The cytoprotective role was associated with a significant upregulation in the activities of key antioxidant enzymes, including SOD, CAT, and glutathione peroxidase (GSH-Px), thereby substantiating their efficacy in mitigating oxidative stress [39]. Conversely, most brown algae exhibited antioxidant capacities below 60%. The sole exceptions included *Odonella aurita*, *Nanochloropsis* sp. and *Bifurcaria bifurcate*. Many studies have highlighted their radical-scavenging and metal-chelating properties; recent research utilizing CTX- and DSS-induced gastrointestinal injury models has demonstrated that marine-derived antioxidant peptides effectively scavenge ROS and diminish oxidative damage in colonic tissues. They also preserve tight junction integrity (e.g., ZO-1, occludin) and promote gut microbiota homeostasis by alleviating dysbiosis and opportunistic overgrowth associated with oxidative injury, ultimately reducing inflammatory signaling pathways (e.g., NF-kB, TLR-4) activated by ROS. These characteristics aid in the prevention of barrier failure, mucosal inflammation, and dysbiosis, all of which are pivotal to the beginning and advancement of colorectal cancer (CRC). The data support the use of marine-derived antioxidants as functional dietary ingredients or adjuvants in the management of oxidative gastrointestinal diseases and the prevention of (CRC) [7,8,9,10,18].

### 3.2. Immunomodulatory Effects

The human immune system plays a critical role in defending against infectious agents and malignant cells via its cellular and humoral components. Its functionality can be compromised by numerous factors, including malnutrition, psychological and oxidative stress, and exposure to external pathogens and antigens. Consequently, nutrition-based interventions, especially functional foods enriched with immunomodulatory peptides, have attracted significant interest due to their potential to beneficially regulate immune responses; they are predominantly derived from marine organisms, including fish, mollusks, crustaceans, sea cucumbers, and marine algae, unless otherwise specified. These marine species represent the principal natural reservoirs of bioactive peptides exhibiting antioxidant, antimicrobial, antihypertensive, and immunomodulatory properties. Although it has been established that these peptides support the immune system by promoting lymphocyte proliferation, enhancing natural killer (NK) cell activity, and regulating cytokines, the precise mechanisms of their action remain unclear [40,41]. Through humoral and cellular mechanisms, the human immune system is essential for controlling and preventing infections and malignancies. The immune system can be weakened by a number of things, such as malnutrition, oxidative and psychological stress, and exposure to exogenous illnesses and antigens. Examples of immunomodulators include actinomycin, vincristine, dexamethasone, levamisole, and Thymosin α1. Nonetheless, their elevated costs and related adverse effects render them inappropriate for prolonged use. Consequently, nutrition-based interventions, particularly functional foods containing immunomodulatory peptides, have gained popularity due to their potential to alter immune system responses. Immunomodulatory peptides have demonstrated the ability to enhance immune processes, including lymphocyte proliferation, NK cell activity, and cytokine modulation. Nonetheless, the mechanisms underlying their operation remain unclear [42]. Marine organisms constitute a significant reservoir of bioactive proteins and peptides with inherent immunomodulatory properties. These bioactive compounds and their derived protein hydrolysates represent promising and sustainable strategies for augmenting overall health and immune regulatory functions. For instance, protein hydrolysates isolated from the skin of the giant croaker (*Nibea japonica*) have been shown to potentiate immune competence by enhancing both cell-mediated immunity, as indicated by stimulated splenocyte proliferation, and humoral immunity, reflected in elevated immunoglobulin levels [40]. Sea cucumbers, particularly those derived from the enzymatic hydrolysis of *Apostichopus japonicus*, are recognized for their immunoregulatory properties. By raising mRNA levels and dose-dependently boosting the availability of NO, TNF-α, and IL-6, hydrolysates improve the immune response. By altering the NF-κB and MAPK signaling pathways in RAW264.7 cells, they stimulate macrophages [34]. In BALB/c mice, oligopeptides produced from the enzymatic hydrolysis of sea cucumbers (*Codonopsis pilosula*) improve humoral and cell-mediated immunity, promote macrophage phagocytosis, and boost NK cell activity [43]. Furthermore, they were shown to potentiate the activity of helper T cells in a C57BL/6 mouse model [44]. Proteins and peptides are interesting prospects for therapeutic development targeted at controlling inflammation, particularly through the manipulation of innate immunity. The creation of therapeutics based on proteins and peptides may lessen the negative immunological responses that are often associated with traditional medicines, while also making the immune system work better [22].

**Figure 2:**
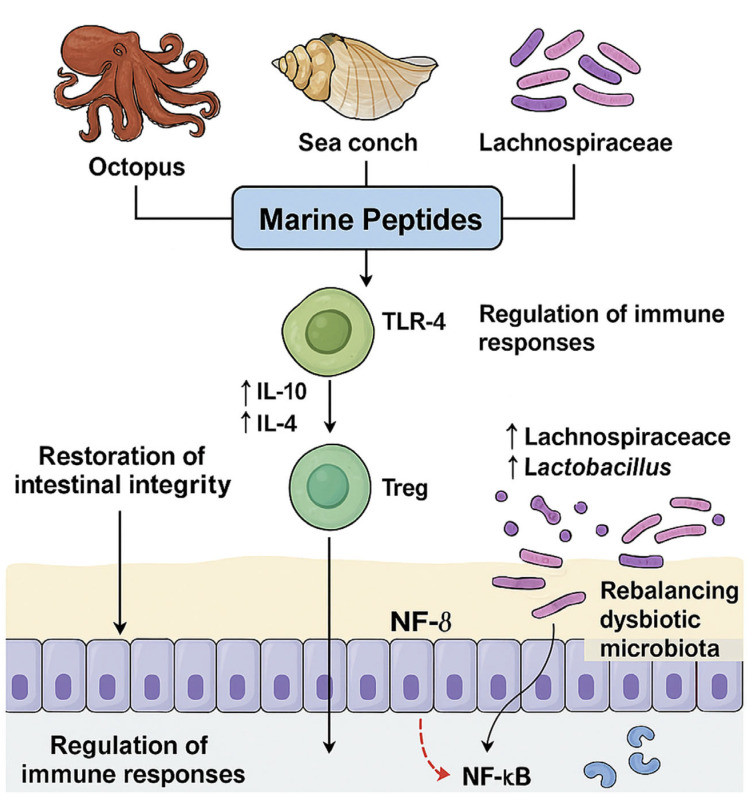
Immunomodulatory mechanisms of marine peptides. Marine peptides activate macrophages, leading to increased production of nitric oxide (NO) and inducible nitric oxide synthase (iNOS), as well as promoting T-cell homeostasis by balancing CD4+ and CD8+ subsets. They downregulate pro-inflammatory cytokines such as TNF-α and IL-1β, while enhancing anti-inflammatory cytokines including IL-10 and IL-4. These immunoregulatory effects are mediated largely through suppression of NF-κB and TLR-4 signaling pathways, resulting in improved immune balance. Upward arrows (↑) indicate increased expression or activity, and the red dotted arrow represents inhibitory regulation between NF-kB and downstream immune signaling components. Overall, marine peptides contribute to restoring immune homeostasis and alleviating inflammation in disease contexts.

The immune system is crucial to maintaining intestinal homeostasis and preventing inflammation-driven conditions such as IBD and chemotherapy-induced gut damage. Peptides derived from marine sources have significant immunomodulatory effects, as they regulate cytokine profiles, enhance immune cell activity, and modulate the signaling pathways involved in inflammation and immune surveillance. A recurrent immunomodulatory mechanism attributed to marine peptides is the homeostatic regulation of cytokine network. Peptide administration consistently results in a dual effect: (1) the suppression of pro-inflammatory cytokines, including tumor necrosis TNF-α, interleukin-1β (IL-1β), and IL-17, which are markedly downregulated in models of colitis and immunosuppression; and (2) the upregulation of anti-inflammatory cytokines such as IL-10 and IL-4, the restoration of which contributes to diminished tissue inflammation and the promotion of immune tolerance. For example, scallop peptide hydrolysates (SCH) significantly downregulated pro-inflammatory cytokines while upregulating IL-10 and IL-4, leading to an overall anti-inflammatory effect in DSS- and cyclophosphamide-treated mice. Marine bioactives influence both innate and adaptive immunity. (1) Macrophage activation: Peptides from octopus, sea conch, and scallop stimulate RAW264.7 macrophages, increasing nitric oxide (NO) production and inducible nitric oxide synthase (iNOS) expression, which are markers of immune activation. (2) T lymphocyte balance: In immunosuppressed mice, SCH and octopus’s peptides restored the ratio of CD4+/CD8+ T cells, promoting adaptive immune balance. (3) Spleen and thymus health: Multiple treatments improved spleen and thymus indices, indicating enhanced systemic immune function [7,8,9,10,18]. Marine peptides influence immune responses by modulating macrophage activation and T-cell balance, as illustrated in Figure 2. Several bioactive compounds play immunoregulatory roles by targeting key inflammatory signaling pathways. (1) Suppression of the NF-kB pathway: LSP, sea conch peptides inhibited the phosphorylation of NF-kB subunits (p65, IkBα, IKK-β), thereby reducing the transcription of inflammatory genes. (2) Inhibition of TLR-4 signaling: these peptides blocked the activation of the TLR-4 pathway, thus attenuating inflammatory responses triggered by microbial or chemical insults. (3) iNOS/NO pathway: SCH treatment enhanced NO production and iNOS expression, indicating heightened macrophage-mediated immune defense. By modulating immune cell populations and cytokine networks, these bioactives help restore immune equilibrium, a critical aspect in both cancer and chronic inflammation. The reduction in CD86 and CD68 expression following LSP and sea conch peptide treatment indicates controlled macrophage activation, reducing excessive immune responses while maintaining defense capacity. In summary, compounds derived from marine sources serve as potent immune modulators, capable of dampening pathological inflammation while preserving (e.g., macrophage activation) and suppressing (e.g., NF-kB/TLR-4 inhibition) immune responses, supporting their therapeutic relevance for immune-mediated diseases [7,8,9,10,18]. Collectively, these findings demonstrate that compounds derived from marine sources can restore immune balance at both systemic and mucosal levels. They achieve this by activating macrophages and lymphocytes, regulating cytokine expression, modulating NF-kB and TLR-4 signaling, enhancing organ-level immunity in the spleen and thymus, and maintaining intestinal homeostasis against inflammatory challenges. These bidirectional immunomodulatory properties indicate their therapeutic potential for preventing CRC, aiding gut injury recovery, and restoring immunity post-chemotherapy.

## 4. Restoration of Intestinal Barrier Integrity

The intestinal barrier consists of mechanical, chemical, immunological, and biological components, each essential for maintaining intestinal homeostasis and preventing external pathogen invasion. Moreover, these components serve as fundamental barriers essential for safeguarding overall health [45]. Collectively, these components constitute a robust defensive system that preserves the homeostasis of the internal milieu and facilitates normal physiological function. The integrity of these mucosal surfaces and their underlying structures can be compromised by external stressors, including chemotherapy, prolonged parental nutrition, malnutrition, trauma, and surgical procedures. Mucosal atrophy, gut microbial dysbiosis, and increased intestinal permeability result from this compromise, leading to intestinal barrier dysfunction. Under typical physiological conditions, the intestinal barrier may fail to function properly if any one of its four components is compromised or abnormal [46]. Intestinal barrier dysfunction increases the severity of pre-existing disorders and promotes the development of new ones. Enterotoxaemia or intestinal bacterial translocation can result from bacteria moving from the intestines to other areas of the body due to a malfunctioning intestinal barrier. Multiple organ failure (MOF) could result from this, continuing the cycle. Therefore, maintaining the intestinal barrier’s functionality and promptly resolving any issues with it are crucial for ending this fundamental cycle and promoting the body’s ability to recover from illness [47]. The physical barrier, which is the mechanical barrier, is the most important part of the intestinal barrier. The mucosal epithelium, lamina propria, and muscularis mucosae make up the physiological structure. Tight junctions, adhesion junctions, and desmosomes are the main types of cellular interactions. Cell connections usually organize intestinal epithelial cells. These junctions keep germs, viruses, and endotoxins from entering into the bloodstream. There are several transmembrane and cytoplasmic-associated proteins that make up tight junctions. Some of these are zonula occludens (ZOs), Claudin, Occludin, and junction adhesion molecules (JAMs) [42]. When these proteins interact with the cytoskeleton, they make complicated structures that act as barriers. Tight junctions have two main biological jobs: they keep the permeability barrier in place, controlling the movement of tiny molecules, ions, and paracellular routes between cells, and they also stop large molecules from entering into cells. Second, they keep cells from moving freely between different compartments by preventing the passage of proteins and lipids. This keeps the balance between the apical and basal cell areas. Bioactive peptides, especially glutamine peptides, have been shown in studies to improve physical barriers and affect tight junctions. By decreasing intestinal permeability and controlling the production of claudin-2 mRNA, the dipeptide alanyl-glutamine has shown the ability to preserve intestinal barrier function in rats following acute, intense exercise [37]. In vivo experiments with piglets have shown comparable outcomes. Evidence suggests that increasing dietary AlaGln levels increases jejunal mucosal protein levels for Occludin and ZO-1 and increases mRNA levels for Claudin-1 and ZO-1. Piglets whose diets include Ala-Gln may have intestinal mucosal barriers that are more functionally intact [48].

The main components of the intestinal chemical barrier are digestive juices, bacteriostatic compounds produced by commensal bacteria, and mucus produced by epithelial cells. A clear mucus layer is created by the intestinal mucosal epithelium’s many goblet cells, which produce a lot of mucus. Molecules from the external environment must first pass through this layer in order to enter the intestine [40]. Musin, which serves as the fundamental structure elements of the mucus layer, is indispensable for preserving the integrity of the colonic epithelial physical barrier [49]: see Musin6 and Musin19. Contemporary research indicates that the compromise of intestinal barrier function induced by mycotoxins is associated with a reduction in mucin secretion [50]. Glutamine-containing peptides contributes to the preservation of the chemical barrier by modulating goblet cell populations and mucin production [48]. Investigations demonstrate that glutamine and alanyl-glutamine can mitigate the suppressive effect of zearalenone on MUC-2 mRNA expression, thereby reinforcing the functional integrity of the intestinal epithelial barrier [51]. Comparable results were documented in a soybean-meal-induced enteropathy model in turbot, where Ala-Gln supplementation significantly upregulated the expression of MUC-2 and ppar-γ genes [52]. In summary, the modulation of mucin expression serves as an effective strategy for protecting the intestinal chemical barrier from injury. The mucosal architecture is characterized by a single layer in the small intestinal and a bilayered structure in the colon, typically encompassing the entire luminal surface of the intestinal mucosa [40]. The outermost layer is loosely structured and teeming with bacteria and their excrement. The inner layer, however, adheres closely to the epithelial cells’ surface and is sterile [53]. The mucus layer keeps symbiotic microbes and host tissues apart from epithelial cells. This prevents harmful substances such as bacteria from penetrating the gut epithelium [54]. The mucopolysaccharide layer consists of mucopolysaccharides, lysozymes, and glycoproteins, which collectively inhibit bacterial growth and colonization.

The biological barrier comprises two principal components: the luminal microbiota and the mucosal microbiota. The luminal microbiota is predominantly populated by bacteria such as *Escherichia coli* and *Enterococcus*, whereas the mucosal microbiota is primarily composed of genera such as *Bifidobacterium* and *Lactobacillus*. These microbial communities adhere to the intestinal mucosal layer, thereby forming a complex, multilayered barrier system [55]. Over a long period of evolution, a stable symbiotic relationship has developed between the flora in the intestines and the environment. The gut gives the body important nutrients and the right conditions for the growth of gut bacteria. The intestinal flora help to lower the permeability of the intestines, boost the body’s defenses, speed up the fermentation of carbohydrates, and create a biological barrier that protects the intestines [56]. The quantity and organization of bacteria within the gut microbiota are typically stable and meticulously regulated. Under standard conditions, the indigenous bacteria in the gastrointestinal tract typically remain non-pathogenic. However, external factors, including immunological challenges or physiological stress, can disrupt the composition and functional capacity of this microbial community. Such disturbances can induce a dysbiotic state, characterized by an imbalance between beneficial and pathogenic bacteria, thereby compromising the integrity of the biological barrier [57]. Alterations in intestinal microbiota composition are associated with the pathogenesis of numerous conditions, including obesity, non-alcoholic fatty liver diseases, inflammatory bowel disease, and other metabolic disorder [58]. Evidenced suggests that glutamine-containing peptides can protect biological barriers by modulating the diversity, evenness, richness, and taxonomic composition of the intestinal microbiota. Specifically, the alanyl-glutamine dipeptide has demonstrated efficacy in mitigating DSS-induced colitis in murine models by remodeling the gut microbiota, notably through a reduction in the Bacteriodetes/Firmicutes ratio and an enhancement of microbial metabolic pathway activity [30]. A previous study showed similar results, showing that glycyl-glutamine protects the intestinal barrier from harm caused by piglet weaning by changing the microbiota in the gut [59]. Adding corn-protein-fermented feed, which is full of glutamine peptides, can significantly improve the gut health of broilers. This feed encourages the growth of helpful bacteria, especially *Lactobacillus*, which makes the intestinal biological barrier function stronger [60]. Furthermore, glutamine peptides have demonstrated a significant increase in the quantity of beneficial bacteria within the intestinal microbiota. Beneficial bacteria primarily generate short-chain fatty acids (SCFAs) as their principal metabolic byproducts. SCFAs are acknowledged for their significant role in maintaining intestinal barrier function through reducing the pH within the intestinal environment. The decrease in pH inhibits the growth of pathogens and induces the expression of tight junction proteins in intestinal epithelial cells. This indicates an alternative mechanism by which glutamine peptides enhance the biological barrier within the intestines [61].

**Figure 3:**
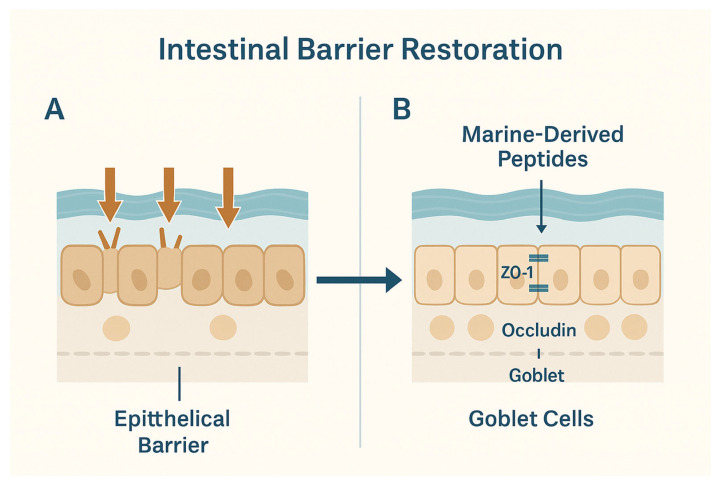
Intestinal barrier restoration by marine-derived peptides. (**A**) The intestinal epithelial barrier is often disrupted in chronic inflammation, leading to increased permeability, microbial translocation, and tissue injury; (**B**) Marine peptides enhance barrier function by upregulating tight junction proteins such as ZO-1 and Occludin, stimulating goblet cell function, and promoting mucin secretion. This strengthens epithelial integrity, reduces microbial infiltration, and protects against toxin-induced damage. Restoration of barrier homeostasis is a key mechanism underlying the therapeutic potential of marine peptides in gut-associated diseases.

The intestinal epithelial barrier is crucial for maintaining gut homeostasis by regulating nutrient absorption, preventing pathogen invasion, and facilitating communication between the immune system and gut microbiota. Disruption of this barrier, often seen in IBD and during chemotherapy, results in increased intestinal permeability, microbial translocation, and chronic inflammation. Marine peptides have been shown to significantly enhance barrier integrity, primarily by regulating tight junction proteins, mucin production, and epithelial structure. Tight junctions are the main structural components that seal adjacent epithelial cells. Several studies have reported that treatment with marine bioactives significantly increased the expression of key tight junction proteins, such as zonula occludens-1 (ZO-1), occludin, and Claudin-1, the restoration of intestinal barrier integrity by marine-derived peptides is illustrated in Figure 3. For instance, scallop peptide hydrolysate reversed the downregulation of tight junction proteins caused by cyclophosphamide or DSS. This restoration is associated with reduced intestinal permeability, improved villus morphology, and overall protection of the mucosal architecture. Mucins are glycoproteins secreted by goblet cells that form a protective mucus layer over the intestinal epithelium. Impaired mucin secretion is a hallmark of barrier dysfunction in colitis and cancer-associated gut injuries. Sea conch peptides significantly increased MUC2 expression, a marker of mucin production. Periodic Acid-Schiff (PAS) and AB-PAS staining confirmed goblet cell regeneration and increased mucous layer thickness in multiple treatment groups. These effects help reduce bacterial adherence to the epithelium and lower immune activation by forming physical and biochemical barriers [7,8,9,10,18].

Histological analysis indicated that treatment with peptides effectively prevented crypt damage, epithelial loss, and inflammatory infiltration. Furthermore, colon length, a key biomarker for colitis severity, showed significant improvement in the treated groups, especially those receiving LSP and scallop peptides. Oxidative stress is known to contribute to epithelial cell damage and mucosal disruption in the intestine. Several compounds with antioxidant properties support the barrier function. These peptides were found to reduce levels of nitric oxide (NO) and malondialdehyde (MDA), both markers of oxidative damage. Enhanced levels of antioxidant enzymes, such as SOD and GSH, were observed in colon tissue, further aiding mucosal healing. The combined effects of tight junction restoration, mucin enhancement, oxidative stress reduction, and histological preservation illustrate that marine bioactives provide multifactorial protection to the intestinal barrier. This positions them as strong candidates for adjunctive therapy in IBD and cancer treatment-related mucosal damage [7,8,9,10,18]. Marine-derived peptides collectively enhance all layers of the intestinal barrier by repairing tight junctions, regulating mucin and goblet cells, modulating cytokine and immune pathways, balancing microbiota, and supporting SCFA. These multifaceted effects make them highly promising functional agents for maintaining intestinal integrity in CRC prevention, IBD management, and post-chemotherapy recovery.

## 5. Gut Microbiota Modulation: Development of New Therapeutics Using Bioactive Peptides

The gut microbiota is particularly important for the health of the host since it impacts digestion, the immune system, the health of the epithelial cells, and the body’s ability to fight off infections. Dysbiosis, marked by an imbalance in microbial composition and activity, is closely linked to IBD, chemotherapy-induced intestinal toxicity, and cancer proliferation. Marine-derived peptides have consistently shown the ability to restore microbial balance, promote beneficial bacterial taxa, and suppress pathogenic species, hence supporting gut homeostasis and systemic immune regulation. The investigation of bioactive peptides has become a crucial pathway for formulating novel treatment approaches, owing to their unique biological properties. Researchers are diligently developing novel therapies that leverage the inherent features of bio-peptides to address various health issues [62]. The angiotensin-converting enzymes (ACE) inhibitory capacity of bioactive peptides has been evaluated in vivo through systematic blood pressure monitoring in spontaneously hypertensive rat (SHR) models. Various administration routes, including intravenous and intraperitoneal injection alongside oral gavage, have been employed to assess the antihypertensive effects of these peptides. Bioactive peptides derived from dietary sources are proposed as natural alternatives for blood pressure regulation, potentially avoiding adverse effects associated with conventional ACE inhibitor drugs. Their mechanism of action involves the inhibition of the enzymes responsible for vasoconstriction, thereby inducing a blood pressure-lowering effects [63]. Chen et al. [57] determined that peptides are capable of traversing the intestinal membrane through various mechanisms, including active transport, passive transcellular diffusion, transcytosis, and tight junctions. It is expected that the absorption of intact lactotripeptides in clinically significant amounts across the intestinal barrier and their later incorporation into the bloodstream will be limited. Single amino acids, dipeptides, and tripeptides are known to effectively traverse the brush border membrane. Prior to entering the bloodstream, the majority of dipeptides and tripeptides undergo hydrolysis into individual amino acids through the action of various digestive enzymes [64]. Some small peptides might be able to move through the brush barrier and into the bloodstream, but the amount is likely to be very small. Thus, it is very unlikely that these functional peptides will reach their target tissues and have the effects they were meant to have [65].

Bioactive peptides that can kill bacteria, fungus, viruses, and cancer cells are quite effective at doing so. Their effectiveness is corroborated by a unique mechanism of action that hinders the development of bacterial resistance [66]. These peptides are very important because they can stop the growth of biofilms. Biofilms are complicated groups of microorganisms that stick to surfaces and avoid immune responses and regular medications. These biofilms often cause long-term illnesses that are linked to medical equipment, making it very hard to get rid of them. Some antimicrobial peptides can stop biofilm formation or make already formed biofilm matrices less stable [41]. Antimicrobial peptides have a lot of antibacterial actions, but one of their most important benefits is that they are not very harmful to mammalian cells. This characteristic makes them an attractive therapeutic option for treating infections, as they may effectively target germs while minimizing harm to humans. However, the utilization of antimicrobial peptides as clinical antimicrobial agents is still in its infancy, and it is essential to address the challenges that hinder their transition from potential to integration into current clinical practices [67]. There are many good things about casein and phosvitin phosphopeptides. The fact that they can bind minerals makes it easier for the body to absorb important minerals including calcium, iron, and magnesium [68]. The intersection of science and health offers a valuable opportunity to explore the potential of casein and phosvitin phosphopeptides in enhancing therapeutic methodologies. Numerous studies have highlighted their anticancer properties, attributed to their ability to safeguard cells from the harmful effects of ROS, such as hydrogen peroxide, superoxide anion, and hydroxyl radicals. While this protective effect is effective in cell culture, its implementation in animal models poses a significant challenge [69]. The application of these results in the development of new pharmaceuticals presents significant potential for advancing cancer treatment and enhancing therapeutic strategies for cancer management. Similarly, specific peptides have the capability to mitigate damage induced by RNS, including peroxynitrite and nitric oxide, the latter being identified as a highly potent oxidant [70]. ROS and RNS have a crucial role in many chronic diseases. Some of these are diabetes, kidney problems, Parkinson’s disease, heart problems, inflammatory diseases, skin ageing, cancer, heart disease, osteoporosis, and gastrointestinal problems [71]. The potential of bioactive peptides as novel therapeutic agents is becoming increasingly apparent. To fully harness their promise, ongoing research must encompass thorough pre-clinical evaluations and clinical trials to confirm their safety and efficacy in specific medical contexts. As medicine continues to advance, bioactive peptides are expected to play a major role in treating a wide range of health problems and greatly improving human health.

**Figure 4:**
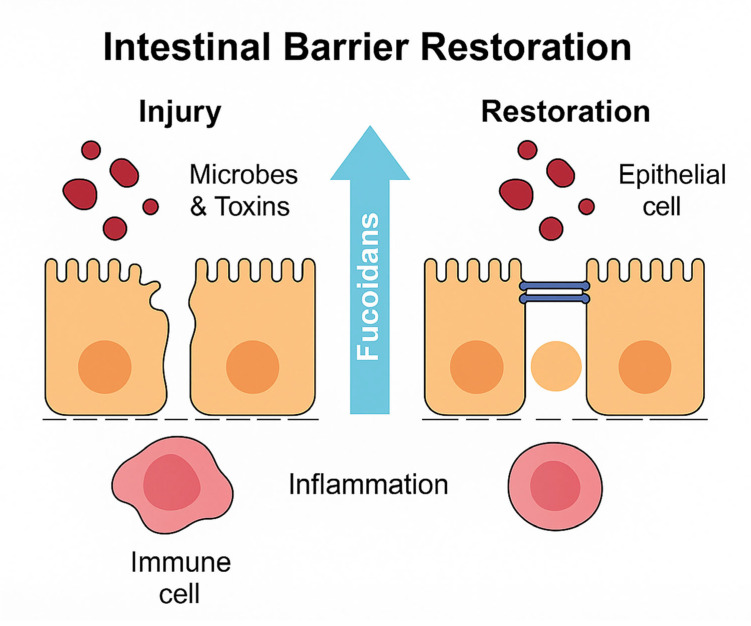
Modulation of gut microbiota by marine peptides. Gut dysbiosis, characterized by the loss of beneficial commensals and expansion of pathogenic bacteria, contributes to intestinal inflammation and tumorigenesis. Marine-derived peptides reshape microbial communities by increasing beneficial bacteria (e.g., *Lactobacillus, Lachnospiraceae*) and reducing harmful taxa such as Enterobacteriaceae. These shifts promote the production of short-chain fatty acids (SCFAs), enhance microbial diversity, and restore ecological balance in the gut. Through these microbiota-regulating effects, marine peptides support intestinal homeostasis and contribute to disease prevention.

In models of colitis and immunosuppression, gut microbial diversity is frequently diminished. However, treatments with marine bioactives have caused significant increases in alpha diversity (the number of different types of microbes in a sample) and beta diversity (the number of different types of microbes between samples). This means that the microbiota ecosystem is more stable and balanced. (1) Treatments with octopus and scallop peptides and LSP raised the number of operational taxonomic units (OTUs) and richness indices, reversing the loss of microbes caused by DSS and CTX. (2) In treated mice, microbial communities exhibited clustering that approximated healthy controls in Principal Coordinate Analysis (PCoA) plots, indicating a reversion to eubiosis. Multiple substances were demonstrated to enhance probiotic and commensal bacterial populations that promote gut integrity and immunological tolerance. LSP notably elevated the *Lactobacillus* and *Lachnospiraceae* NK4A136 genera, linked to short-chain fatty acid (SCFA) production, epithelium healing, and anti-inflammatory properties. Treatments with sea conch stimulated the proliferation of SCFA-producing and mucin-degrading bacteria that confer advantageous functionalities. In addition to probiotic enrichment, these bioactives consistently inhibited colitis-associated bacterial genera, such as Bacteroides, Enterobacteriaceae, and Escherichia/Shigella, which are frequently elevated during active colitis and associated with epithelial damage, barrier disruption, and immune activation, the modulatory effects of marine-derived peptides on gut microbiota and epithelial restoration are illustrated in Figure 4. Their decline in treated groups signifies a transition towards a more favorable microbial ecosystem. Using techniques such as KEGG, FAPROTOX, and LEFSe for metagenomics functional profiling showed that these treatments changed not just the taxonomic composition but also the microbial metabolic pathways. Downregulated pathways included those associated to lipopolysaccharide manufacture, oxidative stress, and pro-inflammatory metabolism. On the other hand, pathways that helped gut repair and immunological balance, such as glucose metabolism, amino acid biosynthesis, and SCFA generation, were enriched. Because the gut microbiota and the immune system are so closely linked, the fact that these bioactives can change microbial communities suggests that they could have a wider therapeutic effect, such as boosting anti-tumor immunity, lowering the toxicity of chemotherapy, and stopping secondary infections. This triangular interaction among microbiota, immunology, and barrier function reinforces the justification for employing marine-derived chemicals in integrated treatment strategies.

## 6. Potential Application in Disease Models

Preclinical studies robustly support the therapeutic potential of peptides sourced from marine environments, underscoring their efficacy in diverse models of inflammatory and immune-mediated conditions. These bioactive substances have demonstrated potential in regulating gut microbiota, re-establishing immunological equilibrium, and mending mucosal barrier functions. These pathways are pertinent not just to IBD but also to chemotherapy-induced gastrointestinal damage and maybe cancer immunotherapy. This review article evaluates various substances use the DSS-induced colitis model, a recognized approach that mimics numerous pathological characteristics of ulcerative colitis. Peptides have been shown to considerably lower disease activity, enhance colon histopathology, and restore epithelial integrity. These peptides inhibited pro-inflammatory cytokines, including TNF-α and IL-1β, augmented the expression of tight junction proteins such as ZO-1 and Occludin, and modified the gut microbiota, suggesting a complex mode of action. Inflammation-associated pathways, such as NF-kB and TLR-4, were downregulated, indicating a possibility for illness adjustment. The intricacy and chronicity of IBD highlight the possibility of these natural items as adjunctive therapy or nutraceutical interventions to enhance current pharmaceutical treatments [7,8,9,10,18].

### 6.1. Chemotherapy-Induced Immunosuppression

The human immune system is fundamental for host defense against infections and malignancies, operating through coordinated cellular and humoral mechanisms. Its efficacy can be compromised by various factors such as malnutrition, psychological and oxidative stress, and exposure to external pathogens and antigens. Consequently, nutritional interventions, particularly functional foods incorporating immunomodulatory peptides, have attracted significant interest due to their capacity to regulate immune functions, including lymphocyte proliferation, natural killer (NK) cell activation, and cytokine modulation, although their precise mechanisms of action require elucidation [72]. Chemotherapeutic agents such as cyclophosphamide (CTX) are acknowledged for causing gut mucosal injury, systemic immunosuppression, and microbial dysbiosis. Marine peptides, especially those derived from scallops and octopuses, have been investigated in CTX-induced immunosuppressed mouse models. These studies show that these peptides can bring back the immune organ indices (spleen and thymus), increase the phagocytic activity of macrophages and the production of nitric oxide (NO), raise the levels of immunoglobulins (IgM and IgG), change the gut flora, and make the intestines more permeable. These results suggest their potential importance in enhancing the immune system during cancer therapy, making them compelling candidates for inclusion in functional foods or adjuvant formulations to mitigate chemotherapy-induced harm.

### 6.2. Implications for Cancer Therapy Support

The reviewed study did not primarily concentrate on the direct anticancer effects of these compounds. However, their capacity to restore gut microbiota balance, enhance immune surveillances, reduce oxidative stress, and improve intestinal barrier function makes them pertinent for strengthening anti-tumor immunity and improving the quality of life in cancer patients. The immune-modulating effects, specifically T-cell rebalancing and macrophage activation, may complement immunotherapies; however, this necessitates validations in tumor-bearing mice. Cancer denotes a collection of diseases marked by the unregulated proliferation of cells and the dissemination of aberrant cells throughout the organism. This syndrome occurs when DNA abnormalities interfere with the usual mechanisms governing cell proliferation and death, resulting in tumor development. Cancer can alter cellular structures and genetic material, and it possesses the ability to metastasize to distant sites within the body [22]. The ability to neutralize carcinogens or inhibit the growth of cancer cells is indicated by anti-carcinogenic, anti-cancer, and anti-proliferative properties. Anti-carcinogens or anti-cancer drugs specifically target the destruction or inhibition of cancer cell proliferation. Bioactive peptides exhibiting anti-cancer activities present prospective therapeutic options by selectively targeting neoplastic cells while minimizing harm to healthy tissues, thus mitigating treatment-related side effects. Flora, fauna, microorganisms, and aquatic species are important sources of bioactive peptides [73]. Furthermore, these peptides inhibit angiogenesis by interfering with vascular endothelial growth factor (VEGF) signaling, consequently restricting tumor access to vital nutrients and oxygen. Additionally, marine peptides demonstrate the capacity to suppress tumor proliferation and enhance the efficacy of conventional cancer treatments through modulation of hyperactive signaling pathways, including P13K/Akt and MAPK [74].

Bioactive peptides, especially those derived from fish hydrolysates, exhibit the ability to mitigate oxidative stress by lowering levels of ROS. The diminishment is crucial for averting genetic modifications, including mutations and chromosomal irregularities, which significantly contribute to the advancement of carcinogenesis. An investigation was conducted to evaluate the anti-proliferative efficacy of protein hydrolysates derived from fish processing by-products against human colon and breast carcinoma cell lines. The study demonstrated that fish protein hydrolysates obtained from the skin, bones, heads, and viscera of multiple species significantly inhibited the proliferation of these malignant cells [75]. The arrangement comprises 33 amino acids organized in an α-helical formation. The mechanism illustrates its potential to address cancer by interrupting the cell cycle at the G2/M phase, consequently impeding the growth of cancer cells [76]. The piscidin family demonstrates significant anticancer properties, particularly piscidin-4, which exhibits pronounced cytotoxic effects on non-small-cell lung cancer (NSCLC) cell lines, including A549, NCI-H661, NCI-H1975, and HCC827. Piscidin-4 induces cell death in NSCLC through a necrotic mechanism instead of an apoptotic pathway [77]. Epinecidin-1, a peptide derived from the orange-spotted grouper (*Epinephelus coioides*), exhibits significant anticancer properties. Research indicates that it can inhibit human lung cancer and glioblastoma by altering cellular mechanisms of survival and apoptosis [78]. Yu et al. [79] conducted a study focused on protein hydrolysates obtained from *Cyclina sinensis* (CSPs) to develop novel pentapeptides that demonstrate anti-proliferative properties, with the ability to induce apoptosis in prostate cancer cells. The findings demonstrated that hydrolysates derived from *C. sinensis* significantly inhibited the proliferation of DU-145 prostate cancer cells. Sponges, which filter feed on particles suspended in the water, produce bioactive chemicals such as peptides that contribute to the mitigation of harmful pollutants in the environment. In the last twenty years, studies have focused on peptides derived from sponges, including jaspamide, koshikamides, and theonellamide G, which exhibit notable cytotoxic effects on various cancer cell lines [80]. These compounds demonstrate cytotoxic effects across a spectrum of cancer cell lines, including breast carcinoma, prostate cancer, acute myeloid leukemia, and colon cancer [81]. The peptides exhibit variation from one another due to the presence of six distinct cysteine residues. The peptides demonstrated significant anti-cancer activity through the inhibition of proliferation in the melanoma cell line A2058 and the human fibroblast cell line MRC-5 [82]. Numerous studies indicate that sea cucumbers may possess properties that contribute to cancer prevention and treatment. A hydrolysate obtained from sea cucumbers demonstrates effectiveness in suppressing the proliferation, migration, and invasion of A549 lung cancer cells. Furthermore, it inhibits the development of pleural effusion, diminishes the proliferation of lung tumors, and prolongs the survival duration of patients with cancer. In C57BL/6 strain mice [83], the peptides, derived from *Cucumaria*, exhibited stability exclusively with PI3K and AKT1. WPPNYQW exhibits considerable promise as an anti-breast-cancer therapeutic, with strong and lasting benefits [84]. Peptides derived from marine sources demonstrate significant anti-cancer potential; however, major information gaps persist that require resolution. These include comprehensive in vivo studies, elucidating particular mechanisms of action, and correlating findings with clinical outcomes. Even though many peptides have been shown to work in vitro, we need to conduct more research on how well they work in people, how stable they are, and how they move through the body. There are significant problems with making medicines on a huge scale, cleaning them up, and making them cheap enough to use. Advanced drug delivery methods, such as nano-carriers, are necessary to overcome these obstacles and enhance peptide stability and targeted distribution. Additionally, using computational methods such as molecular docking and AI-based screening helps speed up the process of choosing peptides for clinical trials. Marine biotechnologists, pharmacologists, and physicians working together will make it possible to move these bioactive compounds from the lab to the clinic.

### 6.3. Translational and Therapeutic Potential Perspectives of Peptides Derived from Marine Organisms with Anticancer Potential

The quantity of natural products is presently on the rise; however, few of these compounds are commercially accessible. Among the extensive collection of peptides derived from marine creatures, only a limited number have undergone comprehensive examination in clinical trials, as outlined in the study by Newman et al., published in 2014 [85]. Certain peptides have been presented as anticancer medicines after successful clinical trials demonstrated their in vivo efficacy. Cemadotin, a peptide sourced from a marine mollusk, and Aplidine, a powerful apoptosis-inducing depsipeptide obtained from the tunicate *Aplidium albicans*, are presently undergoing phase II clinical studies. Kahalalide F, demonstrating potential antitumor efficacy, is currently undergoing phase III clinical studies for the treatment of lung, prostate, and skin cancers [11]. Numerous limitations have constrained comprehensive studies of bioactive peptides sourced from marine creatures. These encompass the insufficiency of adequate chemical amounts, obstacles in sourcing samples, complexities in isolation and purification methods, and environmental problems. Ongoing research continues to explore the promise of marine peptides for numerous medicinal uses. Chemical synthesis is essential for revealing the structure of marine peptides [4]. Nonetheless, the synthesis of enough quantities of these chemicals is a considerable hurdle, hindering the investigation of their biological actions. Moreover, conformational difficulties have been shown to significantly impact the biological function of these compounds. An alternate method for acquiring bioactive molecules with anticancer characteristics involves the enzymatic hydrolysis of marine proteins. The peptides generated using this technique have antioxidant and anti-proliferative properties [86]. By using certain enzymes, it is possible to choose exact cleavage sites in protein sequences, which changes how the resultant peptides work in the body. More research is needed, though, to completely understand the structures of bioactive marine peptides and figure out what they do, such as how they affect the cancer cell cycle. Combining genomics with biosynthesis could be a good way to make marine natural peptides more efficiently and improve their possible medical uses. This combined strategy could lead to new ways to use marine peptides to fight a number of diseases, including cancer. Improvements in genomes, proteomics, and metabolomics have made a significant difference in finding and making peptides that fight cancer. Finding the DNA sequences that code for bioactive peptides is very important for making these molecules. Even though the preclinical results are promising, further research is needed before they may be used in clinical settings. Key areas requiring further advancement encompass the bioavailability and pharmacokinetics of these compounds in humans, the standardization and quality control of polysaccharide and peptide formulations, dose optimization, long-term safety assessments, and clinical trials involving patients with IBD and cancer. These steps are necessary to advance marine bioactives from experimental treatments to medicinal or nutritional treatments that are based on evidence.

**Figure 5:**
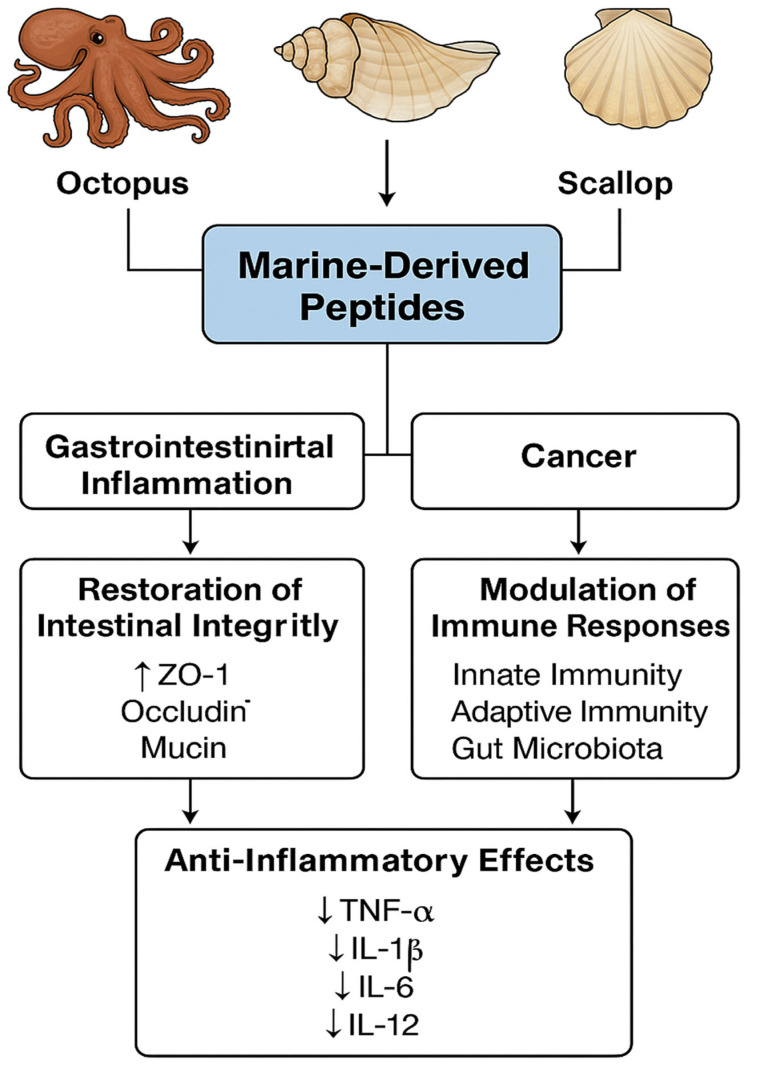
Integrated mechanism of marine peptides in gut health. Marine peptides regulate multiple interconnected pathways to maintain intestinal and immune homeostasis. They inhibit TLR-4 and NF-κB signaling, leading to decreased pro-inflammatory cytokine production and increased anti-inflammatory cytokines. This cytokine balance promotes immune regulation, supports tight junction and mucin synthesis for barrier restoration, and facilitates favorable changes in gut microbiota composition. Upward arrows (↑) indicate upregulation or increased expression, while downward (↓) denote downregulation or decreased expression of the respective molecules. Together, these effects create a synergistic framework in which marine peptides reduce inflammation, restore barrier function, and rebalance microbial ecology, ultimately protecting against IBD and CRC progression.

Marine organisms are important sources of natural bioactive compounds, particularly peptides, which exhibit many therapeutic properties, including immunomodulation, antioxidant effects, and regulation of gut microbiota. These compounds are getting more and more attention since they might help stop and treat stomach problems such as IBD. Peptides derived from marine sources have been shown to enhance immune responses by augmenting the activity of NK cells and raising lymphocyte counts, while concurrently reducing pro-inflammatory cytokines such as TNF-α and IL-6. In murine models demonstrating cyclophosphamide-induced immunosuppression, oral administration of these peptides restored immunological organ parameters and improved gut architecture. They also help maintain the intestines’ health by raising the levels of tight junction proteins and promoting the growth of good gut bacteria. Peptides from marine sources are very good at fighting inflammation and free radicals. These peptides activate macrophages, T cells, and dendritic cells, which changes both the innate and adaptive immune responses. In murine models of DSS-induced colitis, the treatment significantly reduced intestinal inflammation, restored mucosal architecture, and rebalanced the gut microbiota. These effects occur by diminishing TLR-4/NF-kB signaling and elevating the concentrations of anti-inflammatory mediators such as IL-10. Furthermore, these compounds promoted mucin secretion and increased the abundance of beneficial microbial taxa, including *Lactobacillus, Lachnospiraceae*, and *Bifidobacterium*. An overview of the integrated molecules and physiological actions of marine-derived peptides is summarized in Figure 5. Overall, bioactive peptides obtained from marine sources demonstrate considerable therapeutic potential for improving gut health and preventing colorectal cancer by targeting critical components of the immune system, intestinal barrier, and gut microbiome.

## 7. Conclusions and Future Directions

Marine-derived peptides are a promising group of natural bioactive substances that could help with IBD, damage to the intestines caused by chemotherapy, and problems with the immune system. As this review shows, these medicines always show that they can: (1) Change the immune response by controlling pro- and anti-inflammatory cytokines, boosting macrophage activity, and bringing T-cell equilibrium back. (2) Restore the integrity of the intestinal barrier by increasing the production of mucin and tight junction proteins. (3) Improve gut microbiota by adding more beneficial taxa and less detrimental pro-inflammatory species. These effects are mostly caused by blocking important inflammatory signaling pathways including NF-kB and TLR-4 and turning on antioxidant defense systems. These chemicals’ ability to both modulate the immune system and restore gut microbes makes them good candidates for use as adjuvant therapy for gastrointestinal illnesses and as supportive care during cancer treatment. In terms of future outlook, preclinical studies provide significant evidence; however, some essential gaps need to be filled to enable clinical translation. (1) Making extraction and purification processes the same for all batches so that bioactive substances are always the same and can be reproduced. (2) Conducting pharmacokinetic profile and bioavailability studies to find the best ways to give the drug and the right doses. (3) Looking at safety and toxicity in studies that last a long time and involve large doses. (4) Conducting clinical trials in IBD and cancer settings to confirm the drugs’ effectiveness, safety, and patient-reported results. (5) Combining different types of omics to learn more about how host–microbiota compounds interact with each other at the molecular and immunological levels. Overall, the integration of marine bioactives into dietary supplements or therapeutic protocols offers considerable promise for the management of intricate diseases associated with gut inflammation and immunological impairment. Ongoing multidisciplinary research that combines natural product chemistry, immunology, microbiology, and clinical medicine is necessary to fully unlock their therapeutic potential.

## Data Availability

No new data were created or analyzed in this study. Data sharing is not applicable to this article.

## Abbreviations

The following abbreviations are used in this manuscript:

IBD	Inflammatory Bowel Diseases
CRC	Colorectal Cancer
DSS	Dextran Sulfate Sodium
CTX	Cyclophosphamide
ZO-1	Zonula Occludens-1
NF-Κb	Nuclear Factor Kappa B
TLR-4	Toll-Like Receptor 4
IL	Interleukin
TNF-α	Tumor Necrosis Factor Alpha
H_2_O_2_	Hydrogen
MUC2	Mucin 2
LC-MS	Liquid Chromatography-Mass Spectrometry
FTIR	Fourier Transform Infrared Spectroscopy
NK	Nature Killer
NO	Nitric Oxide
SCFA	Short-Chain Fatty Acid
ROS	Reactive Oxygen Species
